# COVI^3^D: Automatic COVID-19 CT Image-Based Classification and Visualization Platform Utilizing Virtual and Augmented Reality Technologies

**DOI:** 10.3390/diagnostics12030649

**Published:** 2022-03-07

**Authors:** Samir Benbelkacem, Adel Oulefki, Sos Agaian, Nadia Zenati-Henda, Thaweesak Trongtirakul, Djamel Aouam, Mostefa Masmoudi, Mohamed Zemmouri

**Affiliations:** 1 Robotics and Industrial Automation Division, Centre de Développement des Technologies Avancées (CDTA), Algiers 16081, Algeria; sbenbelkacem@cdta.dz (S.B.); aoulefki@cdta.dz (A.O.); nzenati@cdta.dz (N.Z.-H.); daouam@cdta.dz (D.A.); mmasmoudi@cdta.dz (M.M.); 2 Department of Computer Science, College of Staten Island, 2800 Victory Blvd Staten Island, New York, NY 10314, USA; 3 Faculty of Industrial Education, Rajamangala University of Technology Phra Nakhon, 399 Samsen Rd. Vachira Phayaban, Bangkok 10300, Thailand; thaweesak.tr@rmutp.ac.th; 4 Department of Computer Science and Information Technology, Kasdi Merbah University of Ouargla, Ouargla 30000, Algeria; zemmouri.mohamed@univ-ouargla.dz

**Keywords:** 3D COVID-19 visualization, voxel-based classification, double logarithmic entropy-based segmentation, virtual reality (VR), augmented reality (AR)

## Abstract

Recently many studies have shown the effectiveness of using augmented reality (AR) and virtual reality (VR) in biomedical image analysis. However, they are not automating the COVID level classification process. Additionally, even with the high potential of CT scan imagery to contribute to research and clinical use of COVID-19 (including two common tasks in lung image analysis: segmentation and classification of infection regions), publicly available data-sets are still a missing part in the system care for Algerian patients. This article proposes designing an automatic VR and AR platform for the severe acute respiratory syndrome coronavirus-2 (SARS-CoV-2) pandemic data analysis, classification, and visualization to address the above-mentioned challenges including (1) utilizing a novel automatic CT image segmentation and localization system to deliver critical information about the shapes and volumes of infected lungs, (2) elaborating volume measurements and lung voxel-based classification procedure, and (3) developing an AR and VR user-friendly three-dimensional interface. It also centered on developing patient questionings and medical staff qualitative feedback, which led to advances in scalability and higher levels of engagement/evaluations. The extensive computer simulations on CT image classification show a better efficiency against the state-of-the-art methods using a COVID-19 dataset of 500 Algerian patients. The developed system has been used by medical professionals for better and faster diagnosis of the disease and providing an effective treatment plan more accurately by using real-time data and patient information.

## 1. Introduction

Variants of COVID-19 have been reported ubiquitously world-wide, causing more infections and spreading faster than any previously known form of the virus [1]. This raises the urgent need for developing effective and safe COVID-19 vaccines [2]. While COVID-19 presents with a variety of symptoms, accurate diagnostic methods are still relevant for slowing the spread of SARS-CoV-2 despite the emergence of COVID-19 vaccines [3,4,5]. Reverse-transcription-polymerase-chain-reaction (RT-PCR) is a commonly used protocol in the detection and quantification of virus infections [6]. However, it is time-consuming and may provide both false-negative (FN) and false-positive (FP) rates [7]. Since COVID-19 causes lung complications, such as pneumonia, computed tomography (CT) of the scan is the most frequently used diagnostic tool [8,9,10]. In Algeria, CT scans have been widely used and show good clinical diagnostics [11]. Both private clinics and public hospital healthcare systems mostly use CT scan imagery to measure the severity of COVID-19 due to the lack of diagnostic kits and higher RT-PCR’s false prediction [12]. Several computer vision applications have emerged to address different sides in the fight against the propagation of COVID-19 [13], including segmentation and severity classification methods [14,15,16].

Since COVID lesions inside patient lungs vary in shape and size, localization of these lesions is still challenging. Segmentation, is a vital step, which allows lesions identification and localization, and separates the lesions from the lung and bronchopulmonary systems. Meanwhile, the lung-region-based methods separate the whole lung (including lobes) from other regions in CT images [17,18]. For the time being, the lung-lesion-based methods split the infected regions from healthy lung regions [19,20,21].

On the other hand, quantification and classification of severity could provide radiologists with relevant assistance for prioritizing patients so that truly serious cases receive care first. Several works have utilized deep learning methods to address the quantification of COVID-19. Shen [22] designed a system that supports radiologists in identifying the patient’s COVID-19 severity degree. Authors in [23] introduced four matrices to evaluate COVID-19-related lesions using chest CT imagery. Work performed in [16] quantified infection by calculating the rates of lesion pixels compared to the lung pixels. Pu, in [24], showed the process of using CT images for quantifying COVID-19 progression and severity in an automated way. Sun [25] developed an extreme gradient boosting (XGBoost) machine learning model for estimating COVID-19 severities by integrating multi-omics data. Finally, Huang [26] described the longitudinal evolution of severity using deep learning methods with CT imagery. The patients were classified into four severity levels and grades (critical, severe, moderate, and mild). The review of the studies showed better performance for severity classification. However, the proposed models failed to take into account small lung regions and regions surrounding the vessels. Moreover, these models often utilized small datasets for training and testing.

Despite great advances in segmentation and severity classification methods for COVID-19 diagnosis, there is still room for additional research to explore these issues. One of the current challenges is to introduce a three-dimensional framework for developing an advanced COVID-19 classification and lesion visualization using cutting-edge technologies such as virtual reality (VR) and augmented reality (AR). In the last decade, researchers in biomedical areas have expressed their interest in VR and AR as novel technologies for better treatments and healthcare information systems (HIS) [27,28,29,30]. This is particularly due to recent developments in camera technology and the processing power of computer hardware. VR and AR could be an alternative solution for the 3D visualization of biomedical images compared to 2D standard technologies [31,32]. They may serve as non-destructive diagnosis methods for better analyzing, locating, and measuring the volume of infected regions [33]. VR immerses users in an entirely digital environment, while AR overlays real-life 3D models. The ongoing COVID-19 pandemic shows an increased need for VR and AR [34,35]. The work presented in [36,37] sets some VR applications related to the pandemic. These cutting-edge technologies could be great for preventing the pandemic. They could also be useful within the health care community.

In this paper, an automated COVID-19 CT-scan imagery data analysis, classification, and 3D visualization using both virtual and augmented reality technologies are reported in detail in the next sections. The main contributions of this paper include the following:

1. A new accurate double logarithmic entropy KL2 algorithm for segmentation and localization in CT images.
2. A novel lesion/lung voxel-based measurement method for quantifying infection.
3. A new combined VR/AR 3D visualization system with a user-friendly interface.
4. A COVI^3^D platform that implements the segmentation, classification and Virtual, Augmented reality algorithms.

This work used a dataset from 500 patients (22,400 CT slices) for tests and validation. The remainder of this paper is structured as follows: Section 2 reports the proposed methods, including the segmentation, 3D classification as well as approaches related to VR and AR visualization and interaction. Section 3 presents experiments and evaluation results. Section 4 and Section 5 provide the discussion and conclusions of the paper.

## 2. Materials and Methods

**Figure 1:**
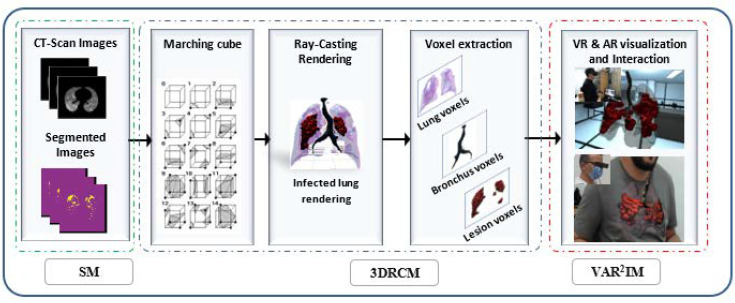
The proposed framework of lesion segmentation, classification and virtual/augmented reality rendering and diagnosis of COVID-19.

A detailed discussion of the COVI^3^D system overview of the proposed method is presented in this section. It consists of three main modules: segmentation module (SM), 3D reconstruction and classification module (3DRCM), and virtual/augmented reality rendering and interaction module (VAR^2^IM), as shown in Figure 1. Firstly, the SM module inputs CT-scan images to get segmental lesions. Secondly, the role of the 3DRCM module is to apply the marching cube and volume rendering algorithms to generate 3D mesh models, and then we introduce the voxel-based classification procedure. Lastly, the VAR^2^IM module provides a volumetric model display of three-dimensional lungs, including lesions.

### 2.1. KL2-Entropy Based Recognition and Segmentation Algorithm

Since there was noise in the input computed tomography images, we pre-processe the data with the algorithm proposed in [38] (see Section 2.1). Once the image was enhanced, we applied a new double logarithmic Kapur entropy (*KL*2) method that partitions a CT image into pneumonia and common regions. The proposed method represents a combination of double logarithmic entropy (*L*2_*Entropy*) and Kapur’s entropy (*K_Entropy*) [39]. L2—entropy is defined as:

(1) $$T_{L}=arg\underset{t}{\max}\left\{s_{1}\left(t\right)\;+\;s_{2}\left(t\right)\right\}$$

where:

$$s_{1}\left(t\right)\;={\left(h_{l}\left(t\right)\;+\;1\right)}^{\gamma }\cos\left(\log\left(\log\left(h_{l}\left(t\right)\;+\;1\right)\right)\right)\;$$

and:

$$s_{2}\left(t\right)\;={\left(h_{u}\left(t\right)\;+\;1\right)}^{\gamma }\cos\left(\log\left(\log\left(h_{u}\left(t\right)\;+\;1\right)\right)\right)\;$$

$$h_{l}\left(t\right)\;=\sum_{S}^{}\sum_{t=1}^{t=n}\left(H\left(s,t\right)\right)\;$$

$$h_{u}\left(t\right)\;=\sum_{S}^{}\sum_{t=n+1}^{t=L}\left(H\left(s,t\right)\right)\;$$

$$H\left(s,t\right)\;=card\left(P_{i,j}\right)\;$$

where *P_i_*_,*j*_ represents a paired image between a given image, *X_i_*_,*j*_, and a denoised image; *H*(*s*, *t*) denotes a matched histogram on a 2D luminance plane, $x_{0}\leq s$ and $t\leq x_{L-1}$. 

*K*_*entropy* is an unsupervised thresholding technique that selects optimal thresholds based on the entropy of segmented histograms [39]. The objective function of Kapur’s entropy can be defined as:

(2) $$T_{K}=arg\underset{t}{\max}\left(K\left(t\right)\right)\;$$

where:

$$K\left(t\right)\;=H_{0}+H_{1}+\cdots +H_{n}\;$$

$$H_{0}=-\sum_{t=0}^{t_{1}-1}\frac{p\left(t\right)}{c_{0}}\log\left(\frac{p\left(t\right)}{c_{0}}\right);\;c_{0}=\sum_{t=0}^{t_{1}-1}p\left(t\right)\;H_{1}=-\sum_{t=t_{1}}^{t_{2}-1}\frac{p\left(t\right)}{c_{1}}\log\left(\frac{p\left(t\right)}{c_{1}}\right);\;c_{1}=\sum_{t=t_{1}}^{t_{2}-1}p\left(t\right)\;H_{n}=-\sum_{t=t_{n}}^{L-1}\frac{p\left(t\right)}{c_{n}}\log\left(\frac{p\left(t\right)}{c_{n}}\right);\;c_{n}=\sum_{t=t_{n}}^{L-1}p\left(t\right)$$

where $H_{0},\;H_{1},\ldots ,H_{n}$ represent the entropy value with $\left\{t_{0},\;t_{1},\ldots ,t_{n}\right\}$ thresholds. *p*(*t*) denotes a probability density function of an image, and $c_{n}$ is a cumulative density function.

*KL2—entropy* is a combination of *Kapur’s threshold* and the *Double Logarithmic* *threshold*:

(3) $$T=\underset{t}{arg\max}\left(\lambda \cdot T_{K}+T_{L}\left(1-\lambda \right)\cdot \frac{T_{K}\cdot T_{L}}{maw\left\{T_{K},T_{L}\right\}}\right)\;$$

where 0 ≤ *λ* ≤ 1.

$T_{K}$ and $T_{L}$ respectively denote *Kapur’s threshold* and the *double logarithmic* *threshold*, and *λ* represents a threshold weight. From the defined region threshold *T*, a local mask of sub-regions was generated as shown in the following equation:

(4) $$m_{i,\;j,\;k}=\left\{\begin{array}{c} x_{L-1}\;if\;card\left(R_{i,j,k}\right)\;\leq \;T\\ x_{0}\;if\;card\left(R_{i,j,k}\right)\;>\;T \end{array}\right.$$

where *R_i_*_,*j*,*k*_ denotes the number of pixels in sub-region *k*. *T* is a threshold. *x*_0_ and *x_L_*_−1_ represent the initial and the last grayscale levels of an image.

To generate the global mask of sub-regions, we rely on:

(5) $$M_{i,j}=\underset{k}{\max}\left(m_{i,j,k}\right)\;$$

where *m_i_*_,*j*,*k*_ represents the local mask of the sub-region and *i*,*j* denotes the size of a given image. *k* is the index of *m_i_*_,*j*,*k*_.

### 2.2. Three-Dimensional Reconstruction and Classification

The surface models of segmented 2D images were used to generate the data volumes by applying iso-surface extraction marching cubes [40] and data volume-rendering [41] algorithms. An iso-surface can be defined as a set of iso-values in a volume data where the expression is given as:

(6) $$\left\{x,\;y,\;z\;\epsilon R^{3}\;:f\left(x,y,z\right)\;=k\right\}$$

where (*x*, *y*, *z*) represents the grid position and k ϵ ℜ represents an iso-value.

The iso-surface extraction process consists of generating triangular meshes to approximately illustrate the desired surface. The marching cubes scheme is essentially based on the divide-and-conquer technique [40] denoting that the volume data is processed by voxels (represented by cubes) [42]. For data volume rendering, we used ray-casting 3D reconstruction algorithm [41]. For each pixel of the screen, the corresponding ray passes through voxels that have been given opacity and color values, thus forming a 3D model that can describe the internal information. For a single voxel, we have two characteristic values [41]: *c*(*x_i_*) is a shade, calculated from a reflection model using the local gradient; *α*(*x_i_*) is an opacity, derived from tissue types of known CT values.

For each voxel along a ray, the standard transparency formula can be defined as:

(7) $$C_{out}=C_{in}\left(1-\alpha \left(x_{i}\right)\right)+c\left(x_{i}\right)\cdot \alpha \left(x_{i}\right)\;$$

where $C_{out}$ describes the outgoing intensity and color for voxel *x_i_* along the ray, and $C_{in}$ is the incoming intensity for the voxel $x_{i}$.

As voxels represent values on regular grids in three-dimensional space, they could be used in refining the classification of infection and to help radiologists discriminate between different severity levels over the dataset.

Let $x_{i}$ denote a voxel of the 3D infected lung model and *n* the number of voxels of the model. The total lung volume is given as:

(8) $$V=\overset{n}{\underset{i=1}{\sum }}x_{i}$$

**Figure 2:**
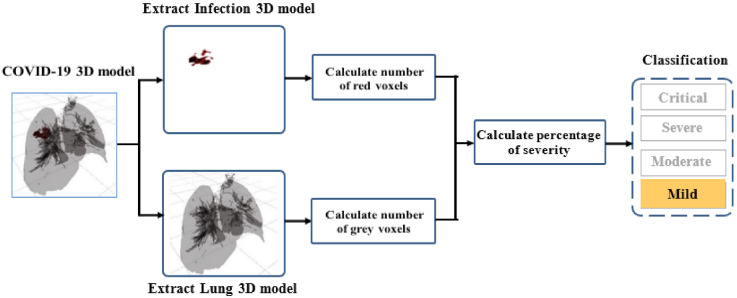
Proposed approach for severity classification.

To quantify the severity, we propose splitting the 3D model into two 3D sub-models. The first sub-model includes the lesion only, while the second sub-model contains lung and bronchi 3D information (see Figure 2). For each sub-model, we calculate the number of voxels as follows:

(9) $$V_{inf}=\overset{n}{\underset{i=1}{\sum }}x_{i\_lesion}$$

(10) $$V_{lung}=\overset{n}{\underset{i=1}{\sum }}x_{i\_lung}$$

where *x_i_lesion_* and *x_i_lung_* represent a lesion voxel and a lung-bronchi voxel, respectively.

Then, we calculate the percentage of infection (*PI*) as the infected volumes $V_{inf}$ over the entire lung-bronchi volume $V_{lung}$. The expression is given below:

(11) $$PI=\frac{V_{inf}}{V_{lung}}\;$$

Figure 2 shows the classification procedure on a 3D infected lung. For better 3D visualization, red color was used for lesion voxels, while grey color was associated with lung-bronchi voxels. The 3D model is classified as positive with COVID-19 when one voxel, at least, is considered as COVID-19 infection. The 3D model contains at least one colored red voxel otherwise, it is considered clean (only gray voxels).

From the value of PI, the patient’s severity was classified (see Figure 2). We applied the approach proposed in [26] to define four classes of severity: mild, moderate, severe, and critical infection.

### 2.3. Virtual and Augmented Reality Visualization and Interaction for Diagnostic Aid

In this sub-section, we integrate both virtual and augmented reality with CT-scan imagery to generate a three-dimensional and realistic display of COVID-19 lesions. Recent research in biomedical applications has shown the usefulness of game engines for 3D medical image visualization [32,43]. The *Unity3D* is one of the prominent game engine platforms (www.unity3d.com, accessed on 16 December 2021) for building 3D medical images and simulation of real-world environments [43]. In our case, we used *Unity3D* as support to generate 3D models.

The dataset contains 3D reconstructed models of infected lungs. *Unity3D* takes charge of several 3D formats, including OBJ and FBX. However, we first converted the 3D reconstructed data into 3D mesh models using *Blender* software. We used decimation to reduce the number of vertices and facets of the 3D meshes. Then, we transformed refined models into FBX datasets overlaid into *Unity3D* as a game component for both AR and VR environments.

#### 2.3.1. 3D Visualization

In terms of VR interface, *Unity3D* encompasses game objects that manage lights, cameras, 3D models and elements of the environment. The most important game objects include 3D lungs models imported into FBX format from *Blender*. To complete the VR diagnostic office, some optional elements could be imported from Unity’s Asset Store and/or dedicated software. VR visualization is performed through a computer monitor connected to a head mounted display (HMD), the standalone Oculus *Quest 2* or *Oculus Rift S*. [44]. Oculus SDK was used as a support for managing both 3D visualization and head and hand movement.

In terms of the AR interface, we integrated *Vuforia* SDK (https://developer.vuforia.com/, accessed on 16 December 2021) for scene recognition and display augmented COVID-19 lungs in the radiologists’ working environment. *Vuforia* supports several functions of *Unity3D*, such as image and video processing, tracking, video rendering, code and user-defined targets. For AR visualization, we utilized Microsoft Surface Tablets and Smartphones, both equipped with a video camera, to process spatial information in three dimensions. We, also, used an AR head display with integrated mono or stereo camera. 

#### 2.3.2. 3D Interaction

We implemented a 3D interaction algorithm through two data-gloves in order to manipulate 3D models in both VR and AR. Two phases were addressed: (1) access the 3D lung and (2) manipulate the 3D lung.

**Access the 3D lung:** we used zoom-in technique [45] for reaching and bringing distant 3D lungs by zooming into the working environment.

**Manipulate the 3D lung:** first, we defined a virtual hand model that reproduces the same movement of a real hand. Then, we calculated the incidence coordinate between two virtual fingers (index and thumb) and a 3D lung. For each position of the two virtual fingers, we updated 3D coordinate lungs to match the hand movements.

Let the encompassing lung volume (E) be $E\subset {ℜ}^{3}$. Let the index and thumb of a virtual hand, be $\left(indexsubset\right)\;\in h$, $\left(thumbsubset\right)\;\in t$ and $\left(h\times t\right)\;\subset {ℜ}^{3}\times {ℜ}^{3}$. Suppose the Volume of the lung (V) where $V\subset {ℜ}^{3}$.

We computed the incidence coordinate between the index and encompassing lung volume (E) as $\left(xd_{i,j},yd_{i,j},zd_{i,j}\right)\;=\;h_{i,j}\subset E$. Then, we computed the incidence coordinate between the thumb and encompassing lung volume (E) as $\left(xt_{k,l},yt_{k,l},zt_{k,l}\right)\;=t_{k,l}\subset E$. 

We calculated the new coordinates of the two incidence coordinates $h_{i+1,j+1}$ and $t_{k+1,l+1}$. Let also $\Theta_{ĩ},\;T_{ĩ}$ be, respectively, rotation matrix, translation vector of a lung *i* along the *x*, *y* and *z* axes.

The manipulation function $M_{lung}\;=\;\left(\Theta_{ĩ},\;T_{ĩ}\right)$ was calculated through the following Algorithm 1:
**Algorithm 1:** 3D manipulation algorithm  **Input:** index’s coordinate $h_{i}$, thumb’s coordinate $t_{k}$, encompassing lung    volume’s coordinates $\left\{e\right\}$  **Output:** lung’s matrix (rotation matrix $\Theta_{ĩ}$, translation vector $T_{ĩ}$)   $h_{i}{}^{T}$ ← *coordinateFormIndex*
$\left(xd_{i},yd_{i},zd_{i}\right)$   $t_{k}{}^{T}$ ← *coordinateFormThumb*
$\left(xt_{k},yt_{k},zt_{k}\right)$   $e_{j}{}^{T}$ ← *coordinateFormCube*
$\left(xc_{j},yc_{j},zc_{j}\right)$   $e_{l}{}^{T}$ ← *coordinateFormCube*
$\left(xc_{l},yc_{l},zc_{l}\right)$  **while** ($h_{i}{}^{T}\neq const$) and ($t_{k}{}^{T}\neq const$) **do**   **if** ($h_{i}{}^{T}=e_{j}{}^{T}$) and ($t_{k}{}^{T}=e_{l}{}^{T}$) **then**    $h_{i,j}{}^{T}$
*← IncidenceIndexCube (*$h_{i}{}^{T}$)    $t_{k,l}{}^{T}$*← IncidenceThumbCube* ($t_{k}{}^{T}$)     **if** ($h_{i+1,j+1}{}^{T}\neq h_{i,j}{}^{T}$) and ($t_{k+1,l+1}{}^{T}\neq t_{k,l}{}^{T}$) **then**      $\Theta_{ĩ}$ ← ($h_{i+1,j+1}{}^{T}-t_{k+1,l+1}{}^{T})\times {\left(h_{i,j}{}^{T}-t_{k,l}{}^{T}\right)}^{-1}\;$      $T_{ĩ}$ ← [$h_{i+1,j+1}{}^{T}]-[\left(h_{i+1,j+1}{}^{T}-t_{k+1,l+1}{}^{T}\right)\times {\left(h_{i,j}{}^{T}-t_{k,l}{}^{T}\right)}^{-1}\times \left(h_{i,j}\right)]$     **end**   **end**  **end**

## 3. Experiments and Summary Results

This section shows the results obtained from the proposed classification and visualization techniques applied on CT-imagery containing 500 with a confirmed positive COVID-19 test. These COVID-19 data were provided from the EL-BAYANE Radiology Center and Medical Imaging and labeled by a medical expert. Furthermore, we provide summary subjective results of the VR and AR visualization and interaction.

### 3.1. Database

**Figure 3:**
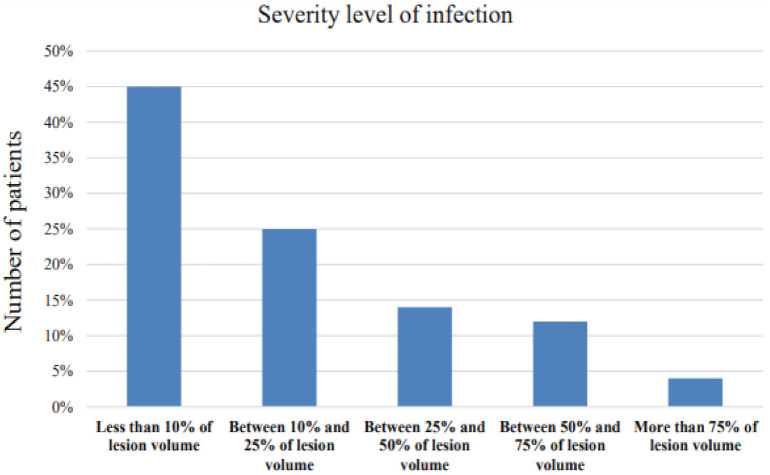
COVID-SVAR data statistics.

Numerous COVID-19 public data-sets are available. However, few of them are from north African countries. Figure 3 shows the statistics of the collected database. In order to obtain high-quality labeled data, we asked two experienced radiologists to tag the maximum number of lesions from CT imagery.

### 3.2. Three-Dimensional Reconstruction and Classification Results

The marching cubes algorithm [40] was applied on segmented images in order to extract polygonal meshes from the iso-surfaces of the 3D pathological structures (conversion from CT voxels to meshes). The meshes were corrected, and consistent models were generated. The voxel scaling effect, due to the segmentation process in anisotropic CT data, was smoothed. Artifacts generated were removed using modifiers such as the *Relax* and *TurboSmooth*, to avoid loss in the model volume. The digital 3D reconstructed models generated can be imported into various digital modeling software that enables good representation, interpretation, and analysis of multi-variant lung involvement with different levels of severity.

We calculate the percentage of infection by the ratio of lesion and lung volumes based on the calculated number of voxels. For example, for a patient with lesion and lung, volume of 273,585 and 6,899,553 voxels respectively, the ratio was 3.96% which corresponds to severity degree *mild*.

We further compared the proposed with an existing classification approach [16] using the same quality metrics (as illustrated in Table 1). We considered the average score of multiple lung region infections.

As illustrated above, the comparison was conducted with four quality metrics. The statistics of the accuracy and specificity were greater using the proposed method. The proposed method showed slightly lower sensitivity and precision values, which may be related to the way the radiologists labeled the dataset.

### 3.3. Virtual Reality, Augmented Reality Visualization and Interaction Results

**Figure 4:**
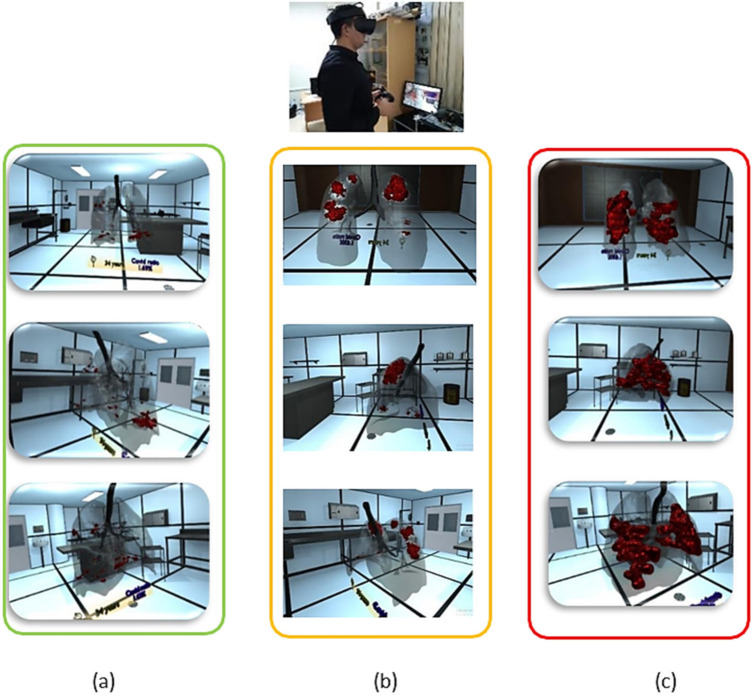
Virtual reality viewer with different stages of disease severity, (**a**) mild, (**b**) moderate and (**c**) critical infection.

Figure 4 shows a radiologist visualizing three situations of COVID-19 severity (mild, moderate, or critical infection) with a VR interface through multi-view access, using an *Oculus Rift S* head mounted display (HMD). The experimenter can, even, navigate into the 3D lungs and see more details on the 3D lesion texture, using touch controllers, such as the developed data-gloves or *Oculus touch*.

The radiologist is capable of making appropriate and quick decisions regarding the three patients. For example, the first patient (see Figure 4a) could be asked to take a treatment and isolate at home, while the third patient (see Figure 4c) would be admitted to the emergency department in the hospital.

**Figure 5:**
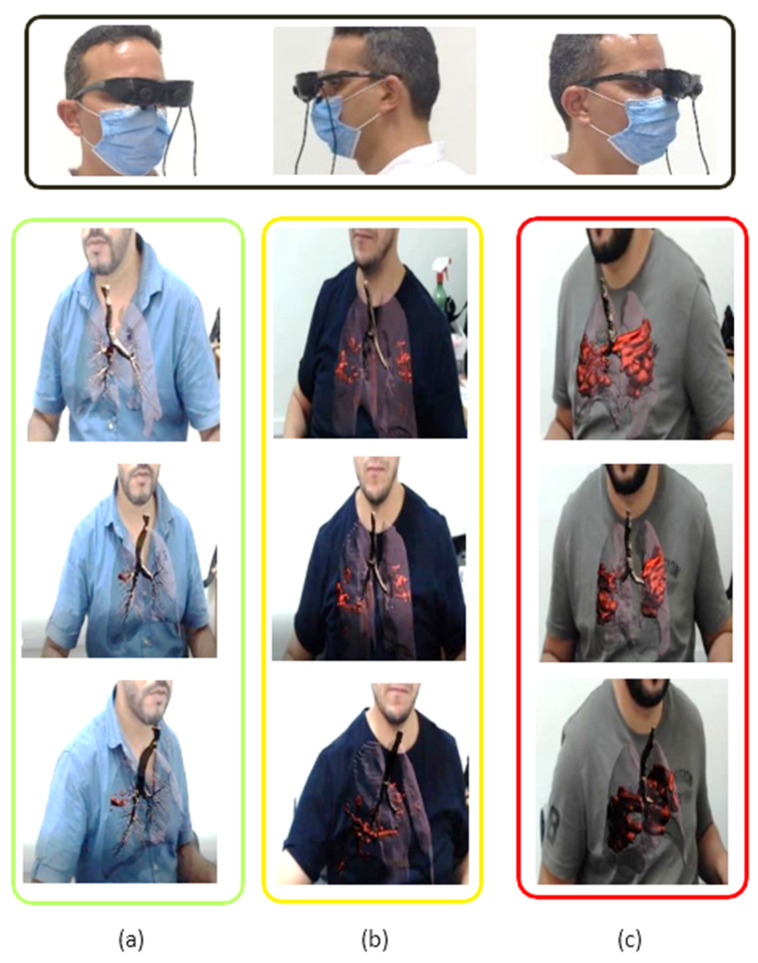
Augmented reality viewer with different stages of disease severity with different patients, (**a**) mild, (**b**) moderate and (**c**) critical infection.

For the AR viewer, we obtained a similar situation, but the 3D models were directly aligned on the patient’s body (see Figure 5). We used a dedicated AR HMD with video camera-integrated and developed data-gloves to process spatial information in the radiologist’s real environment and provide multi-view augmented reality visualization of three patients with different stages of infection (see Figure 5a–c). The radiologist movements, through head and hand tracking, were translated into the 3D working environment, making the experience more realistic.

#### 3.3.1. Experimental Evaluation

We performed a subjective evaluation with more than one hundred COVID-19 infected patients. We compare our proposed visualizations with CT-scan conventional visualizations.

#### 3.3.2. Setup

EL-BAYANE Radiology and Medical Imaging Center provided, for the COVI^3^D platform, an anonymized digital patient database containing CT imagery and patient information, such as diagnostic reports and history. The platform could be generalizable outside of the El-BAYANE Center. COVI^3^D used a workstation MSI-Intel i7-9750H CPU with 2.60 GHz, 32 GB Memory, and Graphic Card NVIDIA^®^ GeForce™ RTX 2060. This workstation was connected with *Oculus Rift™ S*, and *Vuzix’s Wrap 1200* glasses for VR and AR experiences, respectively. The light version of the proposed user interfaces was implemented in Tablets Microsoft Surface with Intel^®^ Pentium^®^ 4415Y CPU with 1.61 GHz, 4 GB Memory, and Intel^®^ HD Graphics 615 graphic card.

#### 3.3.3. Subject

Two main categories of participants were involved in the experiments: (1) recovered patients and (2) medical staff, composed of radiologists/doctors (students, residents and experts) and nurses.

#### 3.3.4. Procedure

(1) Recovered patients (n = 150) were enrolled in this study. The mean age and range were 64.38 ± 7.86 years. We evaluated the awareness of the disease severity and its impact on patients to protect themselves after recovery. The participants were asked to access the diagnosis results through 2D images and 3D models visualized in VR. Each participant had enough time to become familiar with the VR application and CT images. (2) For medical staff, we measured the usefulness of VR and AR as a diagnostic aid. Eighteen subjects (n = 18) experienced both VR and AR applications and provided their feedback. The mean age and range were 48.38 ± 9.32 years. The subjects were instructed to use *Oculus VR* display, *Vuzix AR* glasses, Tablets and Smartphones for two times 8 minutes to be familiar with the 3D interfaces.

Once the experimenters completed their trials, we asked them to fill out two survey questionnaires individually. They responded using a 7-point Likert scale: (1) Strongly Disagree, (2) Disagree, (3) Somewhat Disagree, (4) Fair, (5) Somewhat Agree, (6) Agree, (7) Strongly Agree. Table 2 shows patient questionnaire (PQ), while Table 3 presents the medical staff questionnaire (MSQ).

**Figure 6:**
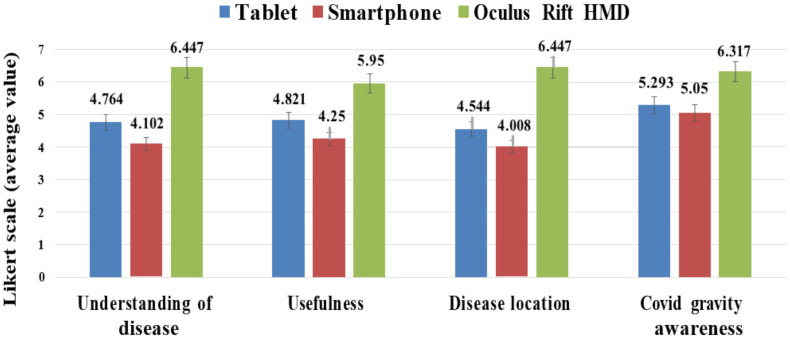
Responses to the PQ questionnaire using the three display methods (the error bars indicate the standard).

**Results:** We analyzed patient and medical staff responses with repeated-measures ANOVAs. Table 4 and Figure 6 show the average responses of PQ regarding 2D images and 3D models and on display devices. Table 5 provides the average responses of MSQ and the differences among residents, experts and students.

**Patient survey:** 37 of 150 patients completed the survey. Overall, the VR models performed better than CT-scan 2D images (see Table 4). Patients provided better comprehension of disease using 3D models compared to 2D images. The same results have been observed regarding lesion size and location. They also noted the best understanding of the treatment plan (6.11/7 vs. 5.03/7). Finally, they reported a greater awareness of the disease severity using VR models compared to CT images.

The 37 participants reported the usefulness of the three display devices (see Figure 6), with results for *Oculus Rift* ranging from 5.95—6.447/7, smartphone from 4.008—5.05/7, and Tablet from 4.544—5.293/7. They found *Oculus Rift* more helpful than Tablet for understanding COVID-19 volume and its distribution inside the lung (6.447 ± 1.043 vs. 4.764 ± 1.988, *p* = 0.03). Moreover, the participants noted that *Oculus Rift* was more valuable than others to be conscious of the severity of disease (6.317 ± 1.302 vs. 5.05 ± 2.345 vs. 5.293 ± 1.435, *p <* 0.04). They provided similar opinions on most questions.

**Medical staff survey:** among 18 participants evaluating the system, we had seven medical students, five radiology residents, three experts and three nurses. Most participants (see Table 5) considered VR and AR experiences as enjoyable (91.32% of responses positive) and agreed that assessment of complex cases was more comprehensive (90.68% of responses positive) and may be performed quickly (81.28% of responses positives). They noticed that AR models provided the true scale of lesion volumes, as well as some pertinent anatomical structures. Participants reported their ability to classify severity cases efficiently and prioritize patients with serious cases to receive treatment first over other patients.

More than 62% rated the didactic potential of VR and AR for clinical use and training residents and medical students. Experts reported the potential for clinical applications and training. Nevertheless, three participants encountered difficulties in being familiar with AR glasses. Two participants complained of motion sickness.

## 4. Discussion

The evaluation results through VR and AR showed the usefulness of 3D display on understanding disease severity. Recovered patients participating in the experiments revealed the potential of VR models in lesion recognition and location compared to 2D CT images. Medical staff found VR and AR enjoyable and easy to use as they can diagnose complex cases better and faster. Moreover, experts pointed out the potential of clinical use of these novel technologies. Finally, residents may benefit from these technologies to take part in the diagnosis. Published studies also show growing interest in using VR and AR to train residents [46,47].

Among the weakness of the study were the motion sickness and individual incompatibility that VR and AR may cause. Appropriate devices, with low latency and high resolutions could further reduce these effects. That irritation could also be minimized through the repetitive use of such technologies.

## 5. Conclusions

Over the last decade, virtual reality and augmented reality have represented a breakthrough for healthcare professionals. They offer 3D models that describe a patient’s internal structure realistically. With the ongoing COVID-19 pandemic, with its variants, the use of these technologies became highly recommended.

The main contribution of this study is to develop an original approach for efficiently bringing together segmentation, classification, virtual and augmented reality with computerized tomography images for COVID-19 diagnostic aid. We provided a powerful automatic platform for visually assessing and classifying the 3D internal lung structure of infected COVID-19 patients to enhance 2D image-based classical diagnostics. Moreover, the platform is able to classify the severity level of COVID-19 based on the Percentage of Infection (PI) through the dataset of more than 500 patients. On the other hand, medical staff reported the usefulness of the proposed COVI^3^D platform in diagnostics since it provides a better interpretation and analysis of radiological results. The proposed could be a serious alternative for treatment planning and training. Doctors expressed their ability to analyze 3D COVID-19 models from different perspectives and with depth recognition, which is not feasible with the 2D screens. However, the 3D models allow better visualization of the giving results with a realistic view and an accurate scale. Thus, we offer the opportunity to save time and money and be able to study the internal textures of infected lungs. Furthermore, the proposed system could also be a relevant solution for recovered patients to be aware of the disease severity and protect themselves and those around them.

In the future, we intend to implement the COVI^3^D platform in public hospitals as well as private clinics to make it easier for doctors and radiologists to review CT scans on a regular basis. As a result, we should continue to advance the development of automatic segmentation, classification, and 3D visualization approaches that may be used in a variety of clinical circumstances. In this work, we only focused on CT medical images, but more research into magnetic resonance compatibility is preferable. COVI^3^D should also be tested on a wide number of medical personnel in a variety of settings, such as ethnicity (Asian, European, etc.). We also plan to extend the platform to support coronavirus mutations and evaluate the robustness of our classification approach against various variants. Finally, we expect to quantify and visualize post-COVID-19 symptoms such as pulmonary embolism following recovery from COVID-19. In the next decade, we think that medical professionals, residents and students could widely use VR and AR as they become available to the general public.

## Figures and Tables

**Table 1:** Quantitative evaluation of severity classification. Bold font indicates best result obtained for each experiment.

Classification of Severity	Accuracy	Precision	Sensitivity	Specificity
Ratio of pixels [16]	0.973 ± 0.02	**0.858± 0.05**	**0.869 ± 0.05**	0.974 ± 0.02
Proposed	**0.996 ± 0.00**	0.796 ± 0.07	0.815 ± 0.06	**0.997 ± 0.03**

**Table 2:** Patient questionnaire (PQ).

Topic	Question
SPQ1: Understanding of disease	How do you rate your comprehension of your COVID/disease? (1: Not at all–7: Very well)
SPQ2: Disease awareness	I understand how big my volume COVID-lesion is? (1: Not at all–7: Very much)
SPQ3: Disease location	I can understand my COVID lesion location (1: Not at all–7: Very well)
SPQ4: Treatment plan awareness	I can understand the reasons my doctor provided the treatment plan? (1: Not at all–7: Very much)
SPQ5: Satisfaction	I’m feeling good with the treatment plan? (1: Not at all–7: Very much)
SPQ6: 3D model analysis	The 3D model helps me to learn about COVID-19 infection? (1: Not at all–7: Very much)
SPQ7: COVID gravity awareness	The 3D model helps me understand the complication from the COVID propagation? (1: Not at all–7: Very much)

**Table 3:** Medical staff questionnaire (MSQ).

Topic	Question
SPQ1: Comfort	Was the VR & AR pleasant? (1: Not at all–7: Very well)
SPQ2: Usefulness (severity classification)	Is the evaluation of complex cases better with VR & AR compared to standard display? (1: Not at all–7: Very much)
SPQ3: Fastness (severity classification)	Is the evaluation of complex cases faster? (1: Not at all–7: Very well)
SPQ4: Training efficiency (1)	How did you rate the ability for student training? (1: Not at all–7: Very much)
SPQ5: Training efficiency (2)	How did you rate the ability for resident training? (1: Not at all–7: Very much)
SPQ6: Practical use	How did you rate the ability for clinical use? (1: Very low–7: Very high)

**Table 4:** Survey responses for understanding of COVID-19 disease using CT scan imagery against VR models.

	CT Images	VR Models
Comprehension of disease	4.670 ± 0.678	6.250 ± 0.494
Lesion size	3.231 ± 0.762	6.193 ± 0.672
Lesion location	3.769 ± 0.525	6.613 ± 0.239
Comfort Level	4.931 ± 0.438	6.108 + 0.219
Awareness of the disease gravity	4.296 ± 0.397	6.201 ± 0.264

**Table 5:** Responses with median answers on the 7-Likert scale.

Questions	Experts (n = 03)	Resident (n = 04)	Medical Students (n = 07)	Nurses (n = 04)
**Comfort**	5.56	6.13	6.43	6.36
**Usefulness (severity classification)**	5.18	6.49	6.58	6.28
**Fastness (severity classification)**	5.10	6.02	6.24	6.10
**Training efficiency (1)**	6.29	6.57	6.71	6.51
**Training efficiency (2)**	6.12	6.21	6.32	6.32
**Practical use**	6.39	6.50	6.61	6.23

## Data Availability

Data supporting reported results can be found at the Centre de Développement des Technologies Avancées (CDTA), Robotics and Industrial Automation Division, Algiers, Algeria.

## Abbreviations

The following abbreviations are used in this manuscript:

COVID-19	Coronavirus Disease
PI	Percentage of Infection
AR	Augmented Reality
VR	Virtual Reality
COVID-SVAR	COVID-19 Segmentation, Virtual and Augmented Reality dataset
HMD	Head-Mounted Display
PQ	Patient Questionnaire
MSQ	Medical Staff Questionnaire
